# Mapping enablers for SDG implementation in the private sector: a systematic literature review and research agenda

**DOI:** 10.1007/s11301-023-00341-9

**Published:** 2023-04-26

**Authors:** Erola Palau-Pinyana, Josep Llach, Llorenç Bagur-Femenías

**Affiliations:** 1 UPF Barcelona School of Management, Barcelona, Spain; 2 Universitat de Girona, Girona, Spain

**Keywords:** Sustainable Development Goals, Implementation, Private sector, Enablers, Systematic literature review, Research agenda, M14, L21

## Abstract

Academics and practitioners alike recognize the important role of businesses in achieving the UN’s Sustainable Development Goals (SDGs). However, research is still needed to understand strategies that can aid the private sector in this regard. The objective of the current paper is twofold. First, it provides an interdisciplinary systematic literature review of 96 papers published between 2015 and 2022 to analyse the state-of-the-art of the academic literature on the enablers that can facilitate SDG implementation in businesses. The analysis provides evidence that enablers can be categorized depending on whether they are external to the company (industry, tools, and education), internal to the company (company characteristics, governance, and adoption of innovation and technology), or a combination of both (Public–Private Partnerships). Second, it provides a specific research agenda on each enabler, offering relevant recommendations for academics, practitioners and policy makers to work simultaneously to achieve the UN’s 2030 Agenda.

## Introduction

The Sustainable Development Goals (SDGs) were introduced by the United Nations in 2015 in the so-called Agenda 2030, as a way to define the global priorities and aspirations at country level to be reached before the year 2030 (UN 2015). The SDGs are a set of 17 ambitious goals that take a broad view of sustainability and respond to the economic, social and environmental challenges faced by the planet. The public sector and governments across the world are already signing up to help achieve these goals (Wynn & Jones 2019). However, recent literature points out that practical challenges for the successful implementation of the goals are understudied, including how the private sector, understood as the ensemble of for-profit corporations that are not owned or operated by the governments, is a vital partner for achieving global sustainable development (Hajer et al. 2015; Kolk 2016; Montiel et al. 2021; Rashed & Shah 2021).

In the same line of thought, several researchers argue that businesses around the world should implement SDGs (Biggeri et al. 2019; Chan et al. 2020; Grainger-Brown & Malekpour 2019), integrate them into their business activities (Biggeri et al. 2019) and report on them (Zemanová & Druláková, 2020). Further, even though the body of literature on the topic has been growing in recent years, sustainable development is progressing slowly (Leleux & van der Kaaij 2019), among other reasons due to the seemingly missing strategies for practical SDG implementation in the private sector (Ferreira Caldana et al. 2022). Alongside this trend, van Tulder et al. (2021) argues that while companies understand ‘why’ they should work to have social, environmental and economic priorities in mind, they lack knowledge as to ‘how’ they should implement the SDGs. Consequently, the support of private companies is not yet fully aligned with countries’ commitments to the SDGs. To counteract this, the aspects that can help firms attain the SDGs must be urgently studied.

Other researchers also emphasize the existing research gaps on how agents with the potential for transformative change other than governments and public sector agents, such as business academics, should adhere to the goals (Hajer et al. 2015; Mio et al. 2020). Studies show that this group is necessary to keep the research field progressing (Christ & Burritt 2019) and, in collaboration with higher education institutions, to train the new generations in alignment with sustainable values, given that training the future business leaders is said to be critical to the success of global SDG implementation (Westerman et al. 2021). Therefore, more academic research is needed, with a special focus on strategies for successful SDG implementation in the private sector because this will ‘provide a long-term conceptual vision, which can interact and converge with practice to better orient business in its role as a sustainable development agent’ (Mio et al. 2020).

With this background in mind, and to extend the present knowledge by means of a new and particular research focus, the present paper aims to provide a comprehensive overview of the aspects that can help implement SDGs successfully in the private sector, so that the recommendations by researchers such as Ferreira Caldana et al. (2022), Mio et al. (2020) and van Tulder et al. (2021) can be accomplished. These aspects are referred to as “enablers”. Hence, the research question addressed in this paper is *What are the current main enablers that can facilitate successful SDG implementation in the private sector?*

To answer this question, we performed an extensive systematic literature review (SLR) addressing the practical implementation of the SDGs in private businesses and focused on identifying the aspects that can facilitate the implementation of the goals. Further, the paper explores how SDG enforcement can aid in the achievement of the 2030 Agenda, developing a roadmap for the future research to be carried out so that the private sector can better achieve the SDGs.

The contribution of the paper is to the recently growing body of literature relating to SDG implementation in the private sector and to the collection and synthesis of available knowledge about the approach companies can take to promote the achievement of the SDGs. The paper does not tackle the public sector because vast research in this regard has already been carried out and governments are already implementing the goals (Wynn & Jones 2019). More specifically, this paper contributes with new insight on the types of enablers that can be used by the private sector to successfully implement the SDGs. It also aims to contribute by providing guidelines for future research on this topic to encourage companies and business academics to pay more attention to the SDGs so that sustainable development can be successful globally. Last, the paper also aims to encourage companies to implement sustainable business activities and to act in alignment with sustainable development.

The structure of the paper is as follows. First, an overview of the research design and process followed is given. Second, the enablers found in the research reviewed are analysed. A future agenda for the research avenues that should be followed in the next decade is then devised, and last, the implications of these findings are discussed and several valuable conclusions shared.

## Methodology

A literature review aims to strengthen the foundation of knowledge on a specific theme by means of synthesizing prior studies and offering a comprehensive review of published research (Paul & Criado 2020). The contributions of reviews of this kind vary depending on the research question, but in general they are directed at (1) resolving ambiguities and outlining the scope of the topic; (2) providing an integrated, synthesized overview of the current state of knowledge; (3) identifying inconsistencies in prior results and potential explanations; (4) evaluating existing methodological approaches and unique insights; (5) developing conceptual frameworks to reconcile and extend past research; and (6) describing research insights, existing gaps and future research directions (Palmatier et al. 2018). Furthermore, according to Snyder (2019), literature reviews can be categorized into three classes, based on the purpose of the article: (1) systematic, (2) semi-systematic, and (3) integrative.

The present paper addresses the practical implementation of SDGs in businesses and aims to find the main typologies of enablers that private corporations can use to promote sustainable development, consequently assisting in the achievement of global sustainable development. Given that the purpose of the article was to synthesize what existing studies show about this particular topic and to provide evidence to inform policy and practice, a systematic literature review was conducted following the guidelines of Snyder (2019).

The methodology to perform the literature review was adapted from Kraus et al. (2020) and Tranfield et al. (2003), who propose that the review is planned in accordance with the following four stages:

- Stage 1: Planning the review, involving the identification of the topic and the development of the search protocol.
- Stage 2: Identifying and evaluating the studies, involving their identification, selection and quality assessment.
- Stage 3: Extracting and synthesizing the data.
- Stage 4: Disseminating the review findings, involving the formulation of the research agenda, the provision of recommendations based on evidence and the creation of the final report to be published.

**Fig. 1:**
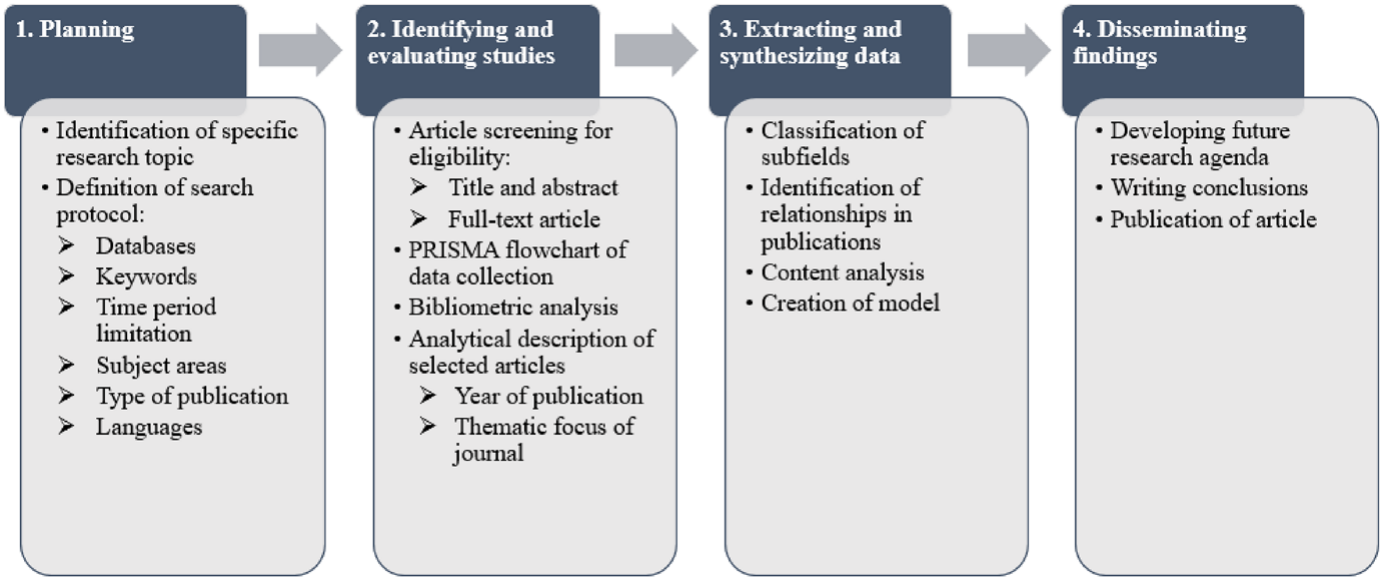
Methodology followed for the systematic literature review. Source: own elaboration based on Kraus et al. (2020); Palmatier et al. (2018); Snyder (2019); Tranfield et al. (2003); van Eck & Waltman (2014)

Figure 1 summarizes the steps followed in each stage.

### Stage 1: Planning

Regarding the first step of the defined methodology, the present article aimed to provide a systematic literature review of the existing academic publications on the implementation of SDGs by businesses to identify the enablers that can facilitate the process.

The objective of a review of this kind is threefold:

1. To develop evidence-informed management knowledge to be used in the promotion and implementation of the UN’s 2030 Agenda,
2. To synthesize the existing knowledge on the topic
3. To identify research gaps for further study.

In terms of the search protocol, the literature search and initial screening were done using two databases: (i) Scopus, the largest abstract and citation database of peer-reviewed literature from Elsevier; and (ii) Web of Science (WoS), a popular classification system by Thomson Reuters. Both were selected with the aim of finding complementary results from high-quality articles published in peer-reviewed journals. The data were extracted on February 08, 2022.

Table 1 summarizes the search protocol.

**Table 1:** Search protocol. Source: own elaboration

Databases	Scopus and Web of Science
Keywords	SDG*; “Sustainable AND Development AND Goal*”; implement*; “Business OR Private sector”
Time period limitation	2015–February 2022
Subject areas	Social sciences, environmental sciences, business, management and accounting
Type of publication	Academic article
Language	English

As the search protocol suggests, we limited the articles to publications issued from 2015 onwards, as this was the year the UN introduced the SDGs. Moreover, articles were limited to the subject areas of social sciences, environmental sciences, business, management and accounting, in English. The key words were chosen on the basis that they were broad enough to capture a diverse set of approaches to the implementation of SDGs in the private sector.

To enhance our understanding of the field, grey literature in the form of practitioner reports, online tools, consultancy reports and conference proceedings were also reviewed. Researchers and experts on SDG implementation were also consulted with the intention of ensuring a wide coverage of the topic.

### Stage 2: Identifying and evaluating studies

**Fig. 2:**
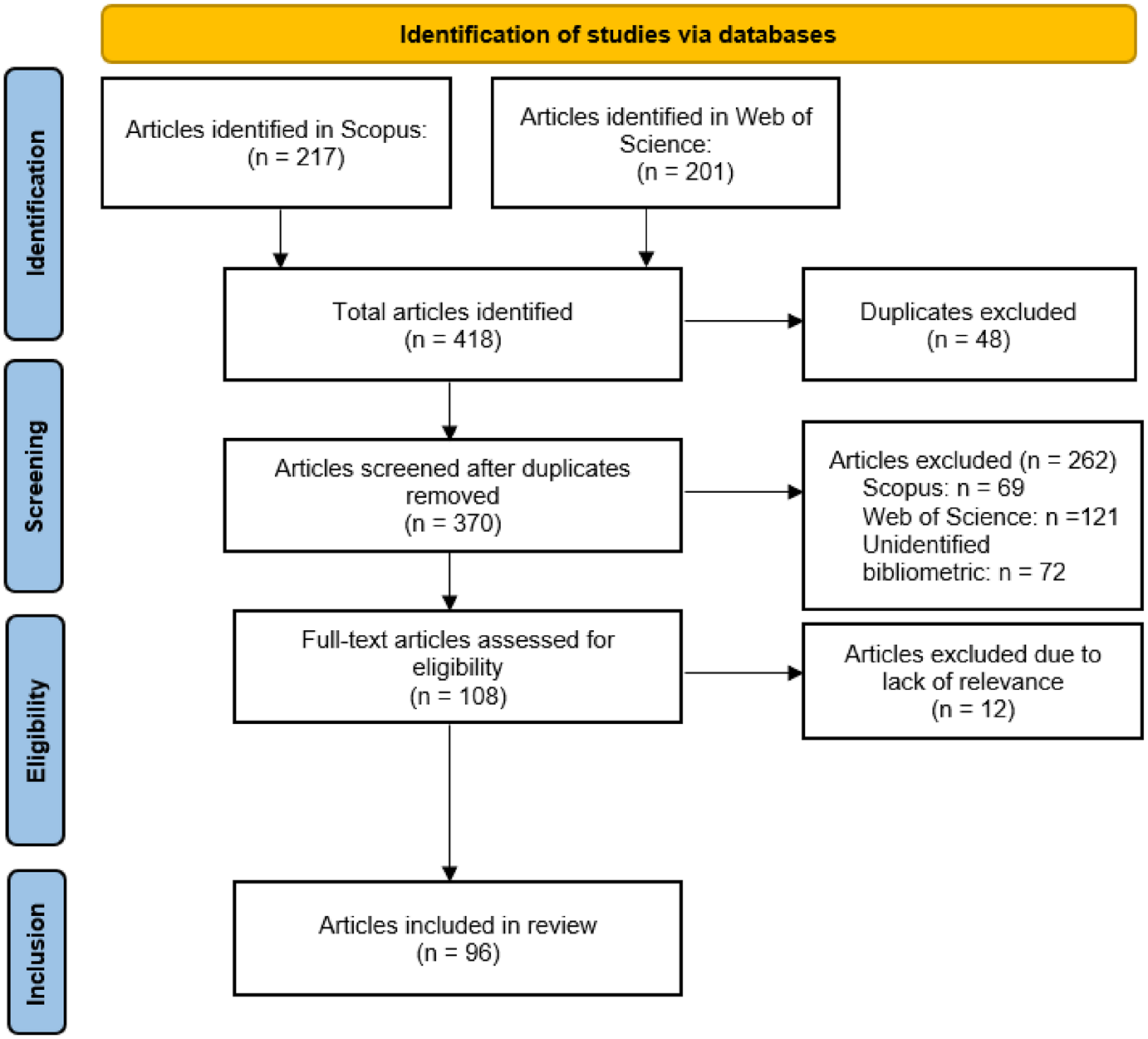
PRISMA flowchart of data collection and selection strategy. Source: own elaboration based on Page et al. (2021)

As summarized in Fig. 2, the literature sample list was refined throughout the research process and some articles excluded, resulting in a final sample of 96 articles. Specifically, from the 418 articles retrieved from the databases, 48 were duplicates and, therefore, discarded. The titles and abstracts of the remaining articles (n = 370) were then screened to determine their relevance for the paper, leading to 262 being excluded for various reasons. The exclusion criteria involved the deletion of articles where the acronym ‘SDG’ did not stand for ‘Sustainable Development Goal’; the deletion of articles that did not revolve around the implementation of the SDGs in the private sector, but rather on their implementation at country level, at an educational level or at citizen level; and the deletion of articles which specifically studied detailed aspects of certain sectors. Seventy-two of the articles excluded in this step were removed due to an unidentified bibliometric relationship, meaning that they had no connection to the rest of the documents. After these exclusions, 108 articles remained to be fully assessed for eligibility.

To map the relationships among the most relevant authors in the literature, a bibliometric analysis of the 108 articles was performed using the open-access software VOSViewer (2021), which showed how the content of the articles related to each other. The output, which can be found in Appendix 1, provides a preliminary overview of the literature. Bibliometric analysis was performed in the second stage of the review because it ‘provides a distance-based visualisation of bibliometric networks in which the distance between two items approximately indicates the relatedness of the items’ (van Eck & Waltman 2014, p.285–320), and also because it facilitates the clarification of subfields and explains the relationships between them by mapping similarities and patterns (Aidi Ahmi 2021; Merigó & Yang 2017; van Eck & Waltman 2014).

The bibliometric analysis in Appendix 1 shows how, despite there being a large amount of overlapping, four clusters can be distinguished. Each cluster is labelled based on its colour (green, blue, red and yellow) and contains the most co-referenced documents, meaning that they mainly deal with similar topics. All titles and abstracts in each group were then simultaneously re-examined so that each field of research could be reviewed. From this analysis, 12 articles were removed due to lack of relevance to the paper at hand. Last, 96 articles were thoroughly reviewed again and included in the content analysis. A table containing the articles included in the analysis based on the cluster they belong to can be found in Appendix 2. All the selected articles fall within the publishing period of 2015 to 2022, with the majority having been published between 2019 and 2021.

The sample of articles was retrieved from 65 different journals with a multidisciplinary scope, with emphasis on the social, economic, environmental and politic activities that occur around the globe. Analysis of the thematic focus of the journals revealed that more than half (55%) of them relate to sustainable development and environmental management, and approximately 13% to management. The remaining third are journals with various subject focuses, such as marketing, higher education and economics, among others.

### Stages 3 and 4: Extracting and synthesizing the data, and disseminating findings

The third and fourth steps of the literature review methodology followed is reported in the analysis of content and conclusion sections that follow.

## Analysis of content

From the bibliometric analysis, four clusters were identified, which revolve around the following four topics:

- Green cluster: guidelines for sustainable development and the achievement of SDGs.
- Blue cluster: international evidence of SDG implementation in different sectors and firm types.
- Red cluster: management education for a sustainable development.
- Yellow cluster: innovative opportunities to achieve sustainable development in the near future.

First, certain academic papers in the green cluster were found to provide instructions to achieve a sustainable development, relating them to the implementation of the SDGs both at corporate and country levels. Second, the blue cluster was found to gather around the analysis of empirical and case studies of companies with miscellaneous characteristics that have adopted the SDGs. Third, the red cluster contained articles on sustainability and managerial education, providing suggestions for business academics. Last, the yellow cluster presented novel alternatives that the private sector could use to assist the progress of sustainable development.

From the analysis of the articles in each cluster, the role of businesses in implementing the SDGs can be understood, the explanation of which can be found in Sect. 3.1. Enablers for SDG implementation were then pinpointed and classified depending on whether they were set in the (1) external environment of the company, meaning that they are a set of exogenous forces that can potentially affect the organization; in the (2) internal environment of the company, referring to the inlying conditions present within the organization; or (3) a combination of the two environments. Additionally, research gaps were identified from the review, and a proposed research agenda can be found in Sect. 3.3.

**Fig. 3:**
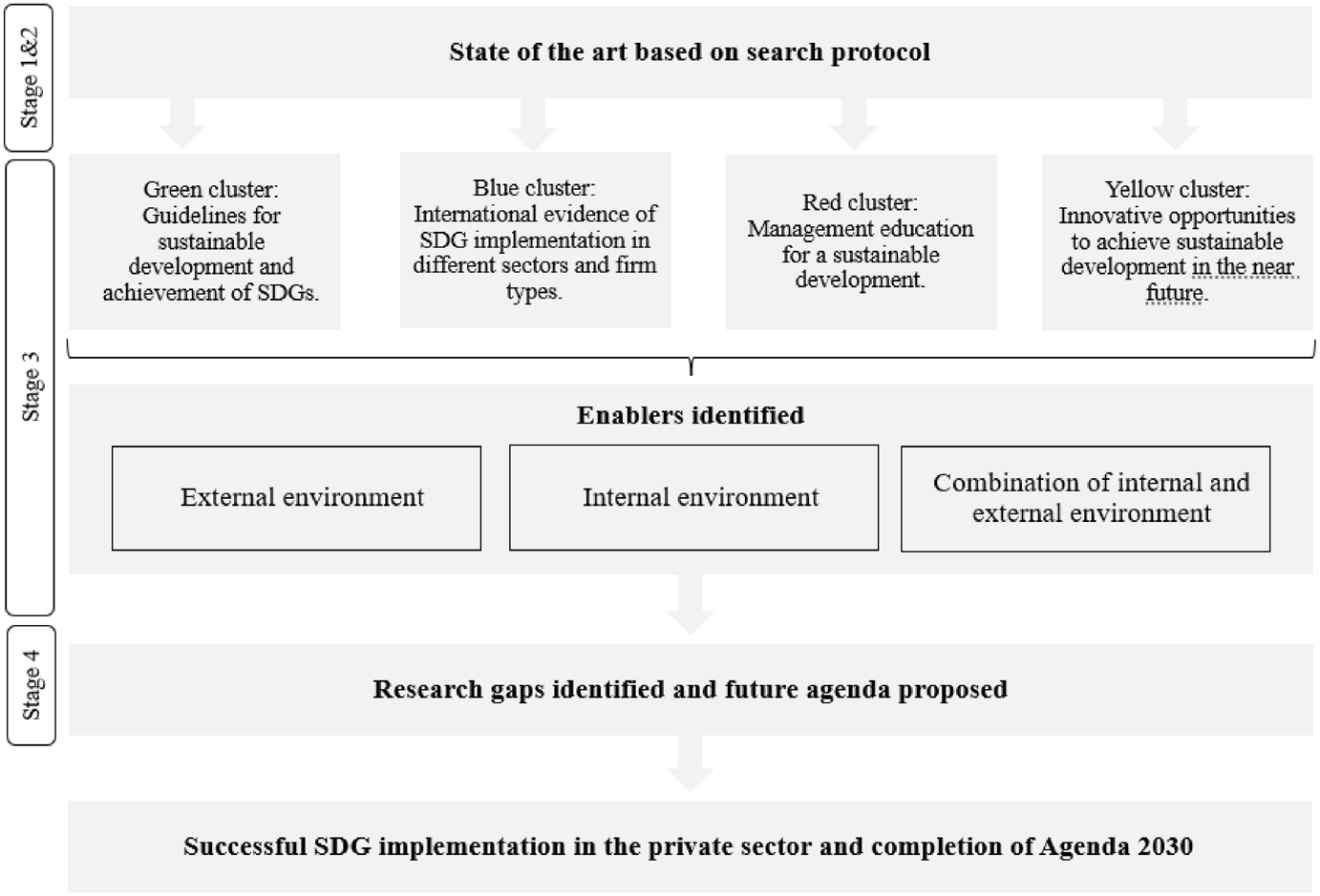
Representation of the content identified in accordance with the methodology stages. Source: own elaboration

The logic behind the content of the literature review is represented in Fig. 3, illustrating that certain enablers, in combination with the future research proposed, are needed to fill the research gaps identified in the literature so that the SDGs can be implemented successfully in the private sector, and the 2030 Agenda achieved in due time.

The content of the literature reviewed is analysed in the following sections.

### The role of businesses in implementing SDGs

Organizations can play a significant role in achieving sustainable development (Rashed & Shah 2021), adopting strategies and practices that support the SDGs (Biggeri et al. 2019) and reporting on them (Zemanová & Druláková, 2020). Kolk et al. (2017) and Montiel et al. (2021) explain that multinational enterprises (MNEs) can play an important role in the implementation of the SDGs as part of their ordinary investments. They can do so by increasing knowledge, wealth and health, and they should particularly aim to reduce negative externalities such as the overuse of natural resources, harm to social cohesion and overconsumption. Liou and Rao-Nicholson (2021) and van Tulder et al. (2021) also acknowledge that MNEs have a vital role in engaging with the goals, which should be implemented in a cross-cutting manner in all the companies’ foreign subsidiaries. Additionally, small and medium enterprises (SMEs) and other for-profit companies can also influence sustainable development, contribute to the environment and aid the country’s economic development. Therefore, all companies across all sectors should devise strategies that consider social and environmental impacts as well as profits (Joseph & Kulkarni 2020). Firms appear to respond positively to the SDGs but critical issues for the successful implementation, understanding and operationalization of the goals in the period up to 2030 are still missing (Sachs et al. 2019; Spangenberg 2017; van Tulder et al. 2021; Wynn & Jones 2019).

On another note, several authors concur that the positive impacts of the implementation of the SDGs in the private sector do not only include the boosting of overall sustainable development but also have advantages at company level. In this line, Jayaprakash and Radhakrishna Pillai (2018) state that the SDGs can be considered a ‘corporate social opportunity’ to obtain a competitive edge over other firms in the industry. This can be clearly seen in the articles by Buhmann et al. (2019), M. J. Santos and Silva Bastos (2021), Yamane and Kaneko (2022), Caldera et al. (2018) and Mattera et al. (2021). Buhmann et al. (2019) state that while implementing the SDGs the private sector can identify opportunities and contribute to the creation of societal value. In a similar vein, M. J. Santos and Silva Bastos (2021) analyse the underlying logic of integrating the SDGs into business management, clarifying that corporate sustainability strategies may leverage the integration of the 2030 Agenda. Yamane and Kaneko (2022) add that the implementation of the SDGs can increase stakeholders’ preferences for companies. Moreover, Caldera et al. (2018) support that engaging in sustainable business practices is a way to improve companies’ sustainability performance, and Mattera et al. (2021) explain that firms that align their activity with sustainability are better prepared to face global crises such as the COVID-19 pandemic. Furthermore, the literature points out that supporting the realization of the SDGs in the private sector also provides opportunities for countries’ entire economies as it can reduce the scale of money laundering activities, which destabilize domestic economies (Dobrowolski & Sułkowski 2020). Notwithstanding, there is some criticism regarding corporate environmental activities, as key stakeholders could regard them as greenwashing. However, Nishitani et al. (2021) refute this assertion by studying the effectiveness of implementing the SDGs in the business targets of Vietnamese companies. Their findings reveal that companies can improve their environmental performance while actively promoting the SDGs.

### Enablers

Certain enablers appear in the literature reviewed as aspects that can aid businesses in successfully implementing the SDGs. The present article provides a classification of these enablers, depending on whether they are (1) external to the environment of the company, meaning that they are a set of exogenous forces that can potentially affect the organization; (2) internal to the environment of the company, referring to the inlying conditions present within the organization; or (3) a combination of both environments.

#### External environment

##### Industry

In their study, Bukalska et al. (2021) suggest that certain contexts and sectors allow for more sustainable activities than others. This is consistent with the findings by the European Environment Agency (EEA 2019) in the report ‘The European environment — state and outlook 2020: knowledge for transition to a sustainable Europe’. The EEA concludes that because of the requirements of each particular sector, the energy consumption and overall sustainability of each differs vastly. For instance, large differences can be appreciated when comparing service and manufacturing industries, as service industries in Europe have a lower energy consumption and lower carbon footprint than manufacturing sectors. The same can be observed with the transport sector, which has a much higher environmental impact than other sectors (EEA 2019).

##### Tools

**Fig. 4:**
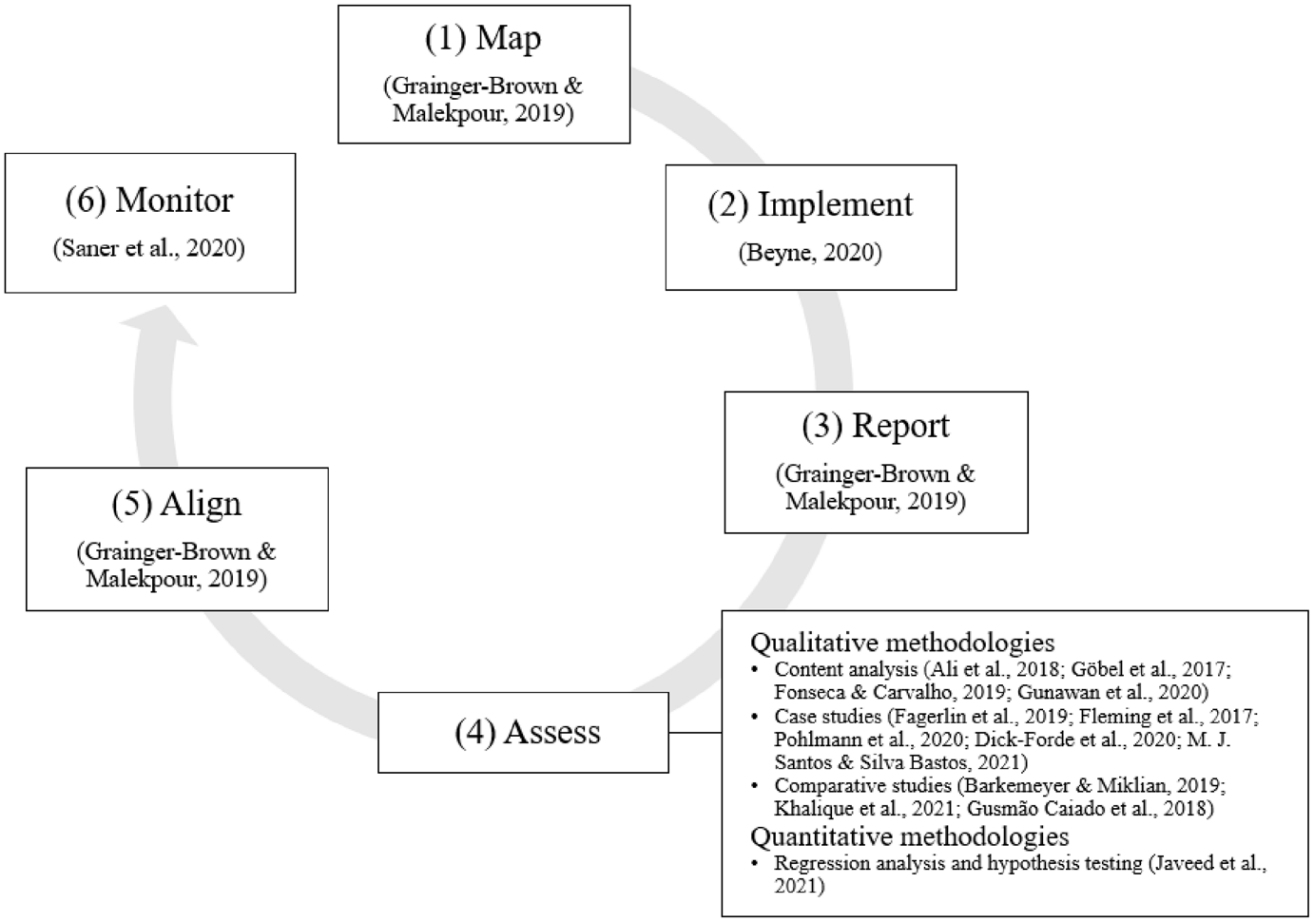
Identified tools. Source: own elaboration based on the reviewed literature

 A myriad of tools and frameworks have been introduced in recent years for companies to engage with the SDGs and assess their implementation. As Grainger-Brown and Malekpour (2019) explain in their scoping review of available frameworks, the thematic typology of tools currently available for organizations to take action towards the achievement of the SDGs revolves around three aspects: mapping of the SDGs, reporting of the SDGs and aligning activities. Moreover, Beyne (2020) complements the proposals of tools by introducing an implementation framework. Further, several studies show the need to assess and monitor SDG implementation. Figure 4 contains the available tools and frameworks identified from the literature review.

Regarding the practical use of the tools, based on the discussion with practitioners, we propose that the order to be followed is: (1) Map, (2) Implement, (3) Report, (4) Assess, (5) Align and (6) Monitor. First, the mapping tools aim to help organizations with evaluating their existing activities related to sustainable development. Examples include the SDG compass, developed by the UN Global Compact (UNGC), the Global Reporting Initiative (GRI), and the World Business Council for Sustainable Development (WBCSD) (2015). The SDG compass is a guide for businesses to take a strategic approach to enhancing sustainable development through core business activities. Another example is the SDG selector developed by PricewaterhouseCoopers (PWC 2016), an online tool that indicates the relevant SDGs for businesses depending on the company’s industry, opportunities they aim to provide, territory and/or theme (people, prosperity, planet, peace or partnership). Another tool is the Mapping to the SDGs (MSDG), developed by the International Capital Market Association (ICMA 2020) to guide public and private sector issuers and investors in reviewing their green, social and sustainability bond issuances and investments against the SDGs. Khaled et al., (2021) also propose a framework to map the SDGs with companies’ Environmental, Social and Governance (ESG) scores, allowing them to identify where the SDGs relate to their ESG performance and to measure their progress towards achieving the SDGs accordingly.

Second, Beyne (2020) has designed an integrative framework of SDG implementation in business strategies, which consists of four stages, the first and foremost of which is ‘inform’. The purpose of this first stage is to get acquainted with the SDGs and understand the opportunities they can represent for the company in building an environmentally and socially responsible future. The second stage, ‘activate’, calls for interaction with stakeholders to identify important sustainability concerns and interconnect them with the guiding principles of the UN’s Agenda 2030. ‘Innovate’ is the third stage, which requires the company to set goals that have the potential to transform the company’s core business for the better. In alignment with this stage, Muff et al. (2018) propose the use of the GAPFRAME methodology to set goals around the following themes: planet, society, economy and governance. This is a planning tool to identify long-term business opportunities and translate the SDGs into indicators for businesses. The fourth and final stage in Beyne’s framework is labelled ‘transformation’, which emphasizes the importance of communicating the changes with key stakeholders, reporting financial and non-financial data and studying the impact of the actions. To communicate the transformative objectives of the company, the mission and vision statements shared on companies’ corporate websites are also deemed as powerful tools (Göbel et al. 2017).

Third, the reporting tools aim to aid organizations in benchmarking activities and communicating progress towards the goals. Examples of reporting tools include the continuously updated Sustainability Reporting Standards, introduced by GRI (2016). This is a set of sustainable principles companies should aim to comply with. It also includes a practical guide on integrating the SDGs into corporate reporting and a document that discloses the GRI standards that can be used to report on specific SDGs. Another guide companies can use was created by KPMG, who launched the document ‘How to report on the SDGs’ in February 2018 (KPMG Report 2018). The report proposes quality criteria for SDG reporting and analyses key statistics on the world’s largest companies so that businesses around the world can benchmark their SDG reporting.

Regarding the assessment of SDG implementation, several qualitative methodologies appear in the literature: content analysis, case studies and comparative studies. Ali et al. (2018) propose the use of content analysis to evaluate the adoption of the SDGs in companies. A similar tool is suggested by Göbel et al. (2017), who regard the mission statement of companies as a means to transfer the SDGs into the sector they belong to, perceiving a well-phrased mission statement as a way to implement sustainable business models. Fonseca and Carvalho (2019a, b) and Gunawan et al. (2020) also perform content analysis to examine annual reports and sustainability reports. Case studies are likewise a tool for describing the implementation of the SDGs in companies (Fagerlin et al. 2019; Fleming et al. 2017a, b; Pohlmann et al. 2020) and commonly involve the use of semi-structured interviews and questionnaires to analyse the priorities and knowledge of the SDGs of key stakeholders (Dick-Forde et al. 2020; M. J. Santos & Silva Bastos 2021). Other analyses are comparative studies of domestic and multinational firms (Barkemeyer & Miklian 2019) and of companies in different countries (Liu et al. 2021). Their aim is to perform benchmarking between companies (Khalique et al. 2021), which can be an important challenge that organizations may face when aiming to study their progress (Gusmão Caiado et al. 2018). As for quantitative methodologies, some studies use regression analysis and hypothesis testing to assess the implementation of the goals in the private sector (Javeed et al. 2021).

Next, ‘aligning’ refers to the tools that can assist organizations in aligning business practices with the SDGs to gain competitive advantage. According to Grainger-Brown and Malekpour (2019), there are few frameworks that aim to align the SDGs in the private sector. However, one such example is the use of the Sustainable Value Exchange Matrix (SVEM), proposed by Morioka et al. (2018), a visual framework to help academics and practitioners discuss sustainable business models and find the resources needed to enable a sustainable value proposition which contributes to the SDGs.

Last, once the SDGs are applied, Saner et al. (2020) suggest that monitoring is necessary to ensure effective and efficient accountability, institutional learning and innovation. They argue, however, that monitoring methods have not yet been defined, leaving businesses without appropriate guidance. Nonetheless, Muntean et al. (2021) state that business intelligence and business analytics can be used to monitor the implementation of the goals.

##### Education

Some of the enablers identified do not concern businesses directly, but rather primary schools and higher education institutions, including business schools and academics themselves, who must work together to reduce the distance between them and practitioners to promote innovative learning materials that involve the SDGs; to offer training that inspires communities to work in alignment with sustainable values (Christ & Burritt 2019; Nakidien et al. 2021); to create awareness among management students (Bhullar 2019); and to develop funding and employment systems that engrain the notion of sustainability into institutions (Son-Turan 2021).

Aleixo et al. (2021) study the importance of universities and business schools in achieving a globally more sustainable future, advocating that these institutions can promote the SDGs in numerous scenarios, such as when carrying out training, research and consulting (de Amorim et al. 2020; Macht et al. 2020; Sierra & Suárez-Collado 2021). In this vein, Pizzutilo and Venezia (2021) argue that policies to enable education for sustainable development in universities should be introduced.

Furthermore, some researchers have conducted studies to support education institutions in the systematic introduction of the SDGs (Alm et al. 2021; Leal Filho et al. 2021), and others have specifically directed their research to training business administration and management students in alignment with sustainability (Gil-Doménech et al. 2021). Moreover, Zanellato and Tiron-Tudor (2021a, b) and Albert and Uhlig (2021) demonstrate the important contribution of higher education institutions toward implementing the SDGs, offering recommendations by means of case analysis studies on Babes-Bolyai University (Romania) and Chemnitz University of Technology (Germany), respectively.

#### Internal environment 

##### Company characteristics

 From the abundant number of organizations around the world that have already implemented the SDGs, it can be observed that several organizational factors are related to the early adoption of SDG reporting and better sustainability practices. According to Rosati and Faria (2019) in their study based on data from 408 organizations worldwide, factors that are related to SDG implementation are a larger firm size, a higher level of intangible assets, a higher commitment to sustainability frameworks and external assurance.

Other researchers concur that entrepreneurship also plays a pivotal role in the sustainable transformation of communities, justifying that through research and development and investment in new technologies and innovation, entrepreneurial projects contribute to the objectives of sustainable development more than larger corporations (Bukalska et al. 2021). In the same study, performed on the Polish market, it was concluded that family firms are more able to contribute to SDGs.

Another aspect with crucial importance for successful implementation is the engagement of the company’s departments, employees and stakeholders. Thus, Westerman (2021) concludes that the Human Resource (HR) department is uniquely positioned to engage firms in cross-functional transformational change efforts.

##### Governance

Firm governance and the attitude of the leaders are other seemingly important aspects of sustainability performance within the company. Rosati and Faria (2019) prove that a higher share of female directors and a younger board of directors relate to a higher level of SDG implementation. Spallini et al. (2021) coincide with Rosati and Faria (2019), correlating the non-financial information in sustainability reports to the presence of female directors and an internal sustainability committee. Bukalska et al. (2021) also point out that certain attitudes, namely aggressive CEO behaviours and CEO overconfidence, can hinder the implementation of the goals.

##### Innovation and technology

In addition, the literature suggests certain enablers related to innovation and technology for businesses to consider. According to Cordova and Celone ( 2019 ), innovation has been proven to be a driver for the implementation of most SDGs, and other researchers agree, adding that responsible business model innovation (Imaz & Eizagirre 2020 ; Taminiau et al. 2017 ), innovative practices such as carbon capture and utilisation (CCU) (Olfe-Kräutlein 2020 ), service innovation (Calabrese et al. 2018 ), and digitalization and digital technology (Camodeca & Almici 2021 ) are aspects that can foster the achievement of corporate sustainable development in the near future.

Popular approaches found repeatedly in the literature reviewed revolve around (1) circular economy (CE) practices; (2) industry 4.0 (I4.0) technologies; (3) sharing economy; (4) cause-related marketing (CRM); (5) bioeconomy; and (6) systems thinking. According to the literature, these approaches have a high potential to contribute to the attainment of different SDGs.

First, CE serves as a solution for many of the negative implications of industrialized sectors, as it can increase efficiency, reduce waste (Ahmed et al. 2021) and improve overall sustainability (Nayal et al. 2021). Evidence of business models integrating CE practices lend support for environmental SDG 6, social SDG 7 and economic SDGs 8, 12 and 15 (Nasution et al. 2020; Schroeder et al. 2019). Additionally, CE is seen as an enabler to also boost all social SDGs (SDGs 1, 2, 3, 4, 5, 7, 11, 16), which should be considered an integral dimension of achieving sustainability (Walker et al. 2021). Moreover, T. Santos et al. (2021) argue that adopting CE practices is a way to prevent the negative effects of the COVID-19 pandemic, which could hinder the adoption of SDGs, especially in developing countries.

Second, I4.0, also known as ‘the fourth industrial revolution’, is the framework upon which modern businesses are designed, emphasizing an increase in interconnectivity and smart automation. The advancement of I4.0 is linked to sustainability potential and can increase value creation, productivity benefits and optimized systems and processes (Habib & Chimsom 2019). It therefore directly affects SDG 9 and SDG 12. Moreover, the combination of CE practices and I4.0 technologies benefit SDGs 3, 7, 8, 9, 11, 12 and 13 (Dantas et al. 2021; Rena et al. 2022). Hence, the nexus has a pivotal role in attaining SDGs (Nayal et al. 2021).

Third, collaborative economy systems aim to share resources among communities to reduce both negative environmental and societal impacts, and costs. These strategies are primarily important in attaining SDGs in the transportation and customer services sectors (Govindan et al. 2020).

Fourth, cause-related marketing (CRM) is another opportunity for businesses and a viable business strategy to implement sustainability initiatives. CRM is the collaboration between a for-profit business and a non-profit organisation, which aims to increase profits and improve society co-ordinately. For instance, Singh and Pathak (2020) state that CRM can support environmental SDGs 7, 12, 13, 14 and 15 in emerging economies.

Furthermore, in certain sectors, important trends that businesses cannot be oblivious to are the bioeconomy and the circular bioeconomy, which have been proven to support innovation, development of sustainable products, competitiveness and cost reduction (D’Amato et al. 2020; DeBoer et al. 2020). In the agriculture and energy industries, for instance, this consists in the use of biomass to produce goods and services (Palmer et al. 2020); and in health-related sectors, in the use of biotechnology. Last, systems thinking can have an important role in the implementation of SDGs because it can enable better conversation and cooperation between agencies (Reynolds et al. 2018).

#### Combination of external and internal environments

##### Public–private partnerships (PPP)

Further to support from within the company, the literature on the role of businesses in attaining the SDGs brings public–private partnerships (PPP) into the picture. Researchers stress the fact that the bridge between policy discussion in global sustainable governance frameworks, such as the SDGs, and practical implementation must be narrowed (Maher & Buhmann 2019). Moreover, cooperation between governments and businesses should be regarded as a tool to guarantee the achievement of the SDGs. Therefore, meaningful multi-stakeholder engagement is needed for their effective implementation (Eweje et al. 2020; Maher & Buhmann 2019; Maslova 2020).

Even though the importance of such partnerships has been growing in recent years, mostly as a government–business nexus in the US and Europe (Bull & McNeill 2019), the considerations of key stakeholders and the SDGs are not always aligned which, in turn, hinders the achievement of the goals (Anderson & Ratiu 2019; Bull & McNeill 2019). Bremermann et al. (2019) exemplify the situation in the Brazilian context, while denouncing the lack of implication of MNEs to accomplish the SDGs and the disregard for the importance of public–private partnerships (PPPs) in accomplishing not only the goals set by the UN, but also in alleviating poverty in the country.

On another note, cross-sector partnerships are also fundamental when implementing the SDGs. For instance, Castillo-Villar (2020a, b) relates PPPs to SDG 17 (on partnerships for the goals), recognizing the essential role of businesses, civil society and universities working together to reach sustainable development. To increase the international performance of these partnerships, Fowler and Biekart (2017) pinpoint the importance of interlocutors, and Yiu and Saner (2017) emphasize the essential role of business diplomacy to ensure companies make an effective contribution to the SDGs and sustainable development. Moreover, Maher and Buhmann (2019) suggest that businesses should engage more with bottom-up approaches where affected groups lead initiatives of sustainability, not only for the completion of the SDGs but also to improve their own lives.

**Fig. 5:**
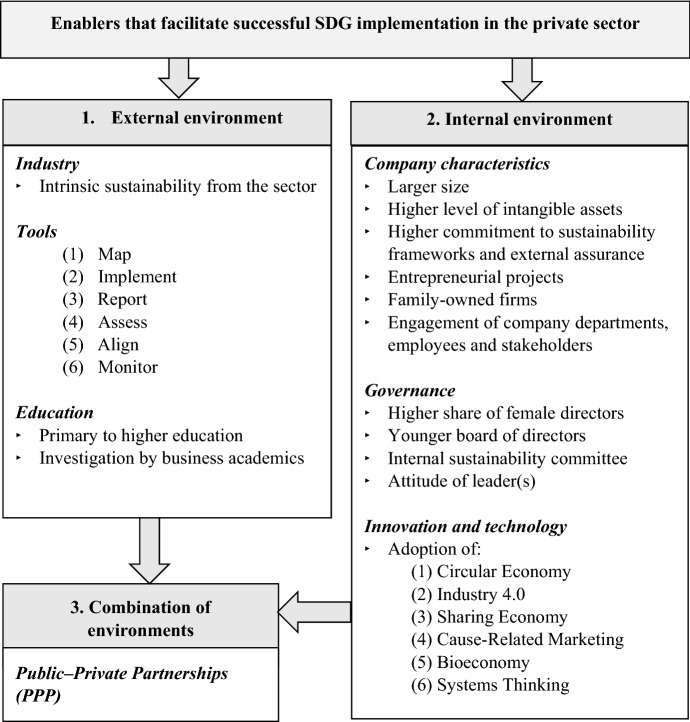
Classification of enablers. Source: own elaboration based on the reviewed literature

Ordonez-Ponce et al. (2021) also recognize the value of sustainability partnerships among organizations in the same area, including Local Agenda 21, among other similar plans. For their part, Sun et al. (2020) indicate that stakeholders from the business, government and non-profit sectors should come together, specifically to address complex challenges such as the environmental SDGs. A good practice in response to climate change is found in Australia, evidenced in Kumar et al. (2020), who perform a qualitative study of 17 businesses with high gas emissions and three industry associations committed to climate action. They conclude that cooperative strategies and top-down legislative measures are necessary to respond to climate change and attain sustainable development. Figure 5 summarizes the identified enablers.

### Future Agenda

Several potential research avenues emerged from the analysis, mostly from analysing the papers’ limitations and future research sections and the authors’ own perspectives. To narrow the identified research gaps in the next decade, useful recommendations can be formulated based on the review, not only for companies but also for governments and education institutions. The proposed methodologies are based on the explanations by Marczyk et al. (2010).

Overall, the literature reveals that the means of SDG implementation in the private sector must be upgraded significantly and shared around the globe for businesses to use. Strategies to better implement the SDGs in the private sector seem to be missing among the articles reviewed. There is a lack of regulated guidelines for SDG implementation, and available plans of action to implement SDGs are needed. Moreover, the literature does not consider the significance of synergetic SDGs, which may be problematic because they are key to attaining the rest of the goals and can help in the progression of others. The goals with the most synergies are SDGs 7 (Affordable and Clean Energy) and SDG 4 (Quality Education) (Boar et al. 2021). Therefore, inductive research to develop theories on the successful strategies of SDG implementation at corporate level should be carried out, emphasizing the research on strategies that deal with the implementation of SDG 7 and SDG 4, since these will lead to the progression of others.

There is a common denominator in the categories of the future agenda, which is the fact that all theoretical research should be followed by applied research. Case studies should therefore be performed continuously, as should empirical studies that analyse the situation in certain regions, allowing the comparison of results among companies and countries. With that said, as (Guillén et al. 2021) indicate, the challenge in this area of research is theoretical in nature. The specific future agenda for each aspect explored in the present literature review can be found below.

#### External environment

##### Industry

 Little information is available on the role of the industry as an enabler to implement the SDGs in the private sector, as few studies consider this factor. Therefore, knowledge should be deepened on the influence of the sector as an enabler for SDG implementation. To put the theoretic knowledge into practice, studies should compare the sustainability performance of companies with similar profiles (in terms of size, assets, governance style, etc.) across different sectors. The studies should be directed at offering recommendations on the best strategies to implement the SDGs in sectors that are not intrinsically sustainable, such as the transportation, manufacturing and extractive industries.

##### Tools

 The present literature review provides a preliminary overview of the currently available tools and frameworks, revealing that they lack standardization, which makes results incomparable and inhibits wider adoption of the SDGs in business strategies. Furthermore, no studies that compare the success rate of using different tools were found, meaning that companies do not know what tools they should use to successfully implement the SDGs. To counteract this, secondary research should be carried out on the tools and frameworks available, considering their success rate when differentiating between sectors, business types and country development level. This research should be complemented with applied experimental research to test the use of available tools in different companies.

From the tools identified through the research review, there is a clear research gap on the available means to align business practices with the SDGs and to monitor the success of SDG implementation within the company. Accordingly, exploratory research should be directed towards the under-researched aspect of SDG alignment and goal monitorization at corporate level to measure the gains brought about by the implementation of the goals, and to report on the results. Furthermore, the introduction, usage and success of key performance indicators (KPIs) related to the SDGs should be studied and complemented with case studies aimed at tracking the success of SDG implementation in the company.

##### Education

 Part of the literature reviewed concerns SDG 4, since it deals with the critical role of universities and higher education institutions in aiding the achievement of sustainable development through the private sector. Nonetheless, business academics should continue investigating in alignment with the recommendations that arise from the present research. The focus of these studies should be on transforming education facilities and their curriculums in alignment with sustainable development. Furthermore, research is limited concerning the future steps companies should take after the goals are implemented in the company, so studies should be carried out towards defining a roadmap for sustainable development after the completion of the 2030 Agenda.

#### Internal to the private sector

##### Company characteristics

 The importance of *how*, *why*, and *which* company characteristics (such as the financial returns, the non-financial situation of the firm and the number of employees) can enable the adoption of the goals is seldomly expressed in the literature. In the proposed research, the company characteristics that allow for a more successful SDG implementation should be defined and proved using multiple case studies. In addition, further research on the implementation of the goals in different types of companies should be undertaken. While there are certain publications on the role of MNEs and SMEs, there is a lack of information on B corporations, which have been argued to actively support sustainable development (Kirst et al. 2021), and on family firms. Therefore, the research focus should be set on case studies and empirical studies to assess the success of SDG implementation among B corporations, and to compare SDG implementation in family firms versus non-family firms to assess whether ownership is related to better sustainability results.

##### Governance

 In the same line, further research is needed on *how*, *why* and *which* corporate governance characteristics (such as a higher proportion of female directors and a younger board of directors) can enable SDG implementation. Following the suggestions of Winschel and Stawinoga (2019), future research should also revolve around how sustainability-oriented CEO compensation can aid in improving overall sustainability. Additional qualitative analysis should also be performed to examine whether other governance details, including managerial styles, corporate organization, transparency and the introduction of sustainability management systems, positively influence SDG implementation.

##### Innovation and technology

Regarding the theme of innovative solutions, the literature review indicates the relevance of exploring the following approaches to enable sustainable development and to tackle harmful environmental impacts: CE practices, I4.0 technologies, sharing economy, CRM, bioeconomy and systems thinking. However, other novel potential solutions should also be explored and subsequently tested across sectors and company types. Furthermore, particular attention should be paid to synergetic SDG 7, since it can aid in the accomplishment of other SDGs.

#### Combination of external and internal environments

##### Public–private partnerships

 Regarding PPP, a better understanding of the implementation of the SDGs in public organizations is needed, as well as more knowledge on the necessary policies that will enable the completion of the 2030 Agenda (Allen et al. 2018). Research is also limited on the role of local administrations in supporting SDG implementation in private businesses, so studies should be performed with this in mind. Additional investigations should be directed towards finding and testing more cooperative strategies to enable SDG implementation, and on defining top-down legislative measures and policies that can facilitate sustainable development. The results of all the proposed studies should be aimed at better policymaking and planning at national and international levels.

**Fig. 6:**
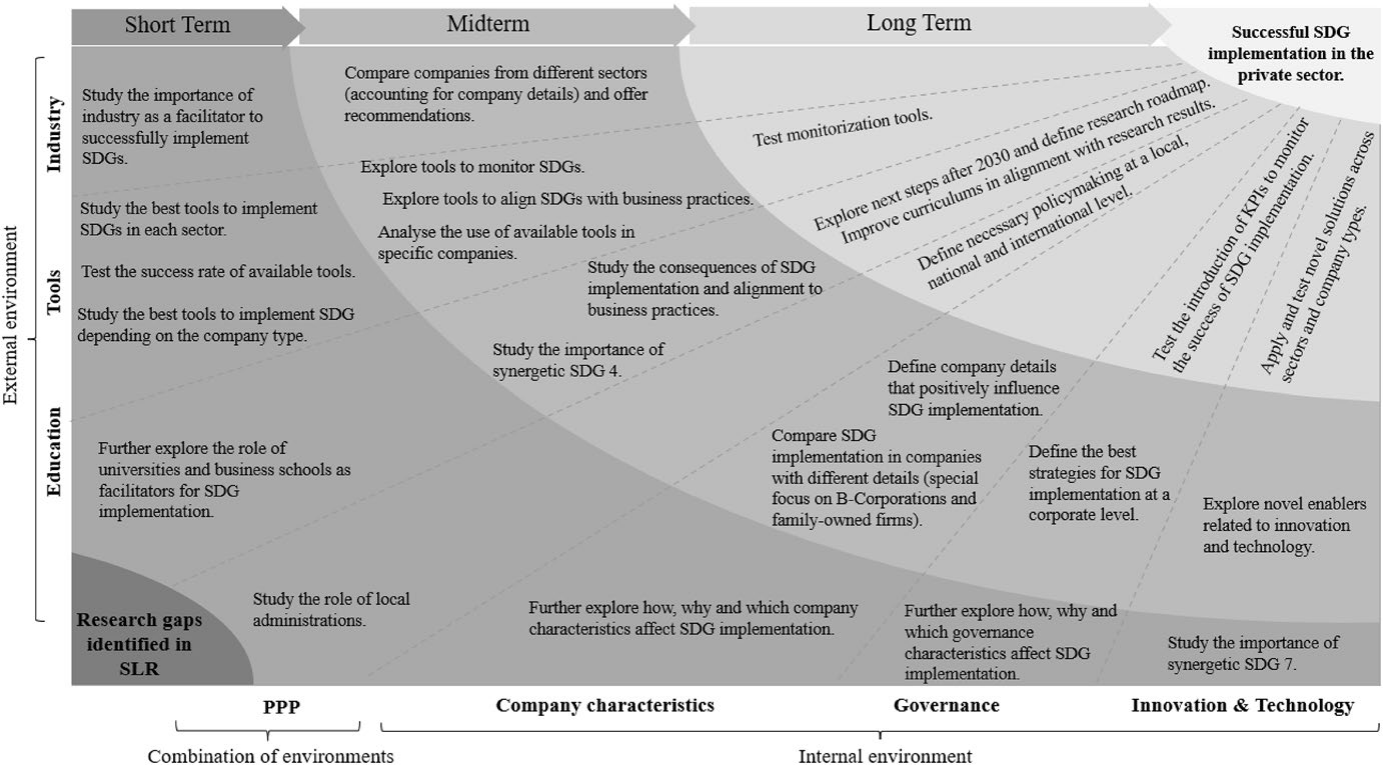
Roadmap for the research agenda proposed. Source: own elaboration

The roadmap for the future agenda suggested is summarized in Fig. 6 and is based on when research is recommended to be carried out. It differentiates between the studies that should be done in the short term (between the years 2022 and 2023), the midterm (between 2023 and 2028) and the long term (between 2028 and 2030).

## Conclusions

Based on a sample of 96 English language peer-reviewed articles from indexed scientific journals, the present paper reviews the relevant literature to answer the research question proposed: What are the current main enablers that can facilitate successful SDG implementation in the private sector?

Overall, the review identified three categories of current enablers. First, those that are external to the environment of the company, meaning that they are a set of exogenous forces that can potentially affect the organization. These include the industry, the available tools needed to thoroughly put SDGs into practice, and education. Second, there are certain enablers that are endogenous to the firm’s environment, which include the company’s characteristics, its governance, and the solutions related to innovation and technology that each company can adopt. Third, a combination of both environments is examined, with public–private partnerships identified as a relevant enabler for SDG implementation.

The paper supports that SDGs have a transformative potential for countries that companies should not disregard, since they can aid in the completion of the 2030 Agenda. Therefore, the private sector should pay special attention to these enablers as strategies to implement the goals because they will lead to improving the firm, building a better economy for the country, attaining environmental sustainability and social inclusion, and minimizing environmental degradation and socio-economic turmoil. This paper provides new insights, highlighting relevant strategies for businesses to contemplate and suggesting future research that should be conducted so that the SDGs can be successfully implemented during the next decade. Furthermore, the review carries significant implications for business academics, practitioners and policymakers.

First, several implications for business academics emerge, as this group has the potential to train the new generation of future business leaders in alignment with sustainable values. According to Aleixo et al. (2021), students are currently keen to learn more about sustainable development, thus the role of professors is a key contribution to sustainable development. Furthermore, business academics can perform management research, implement consultancy activities and enlarge the knowledge base of SDG implementation strategies in their respective countries.

Second, both business directors and employees should understand the importance of implementing the SDGs in their organisations in favour of achieving sustainable development, and developing supportive strategies that enhance social benefits and limit environmental harm while maintaining profits. To accomplish this aim and attain excellence, core competencies at corporate and managerial levels are necessary. Notably, the literature advises companies to be aware of the risk that working towards the implementation of the SDGs could be regarded as greenwashing by stakeholders. To prevent this, the non-financial results and sustainability strategies should be communicated in annual reports, since reporting is an effective way to close the gap between stakeholders’ preferences, companies’ priorities and, in this specific case, sustainable development. Moreover, businesses should work with a multi-stakeholder approach in mind because achieving all the SDGs soon is beyond the reach of any single firm, but rather requires the collaboration of all actors in society.

Third, the review also carries implications for governments, who must be encouraged to provide an understanding of SDGs and to disclose the country’s commitment to goals so that companies can follow their example. Legislation aligned with the SDGs should also be introduced, with governments thereby providing guidelines for SDG implementation. Several authors point out that governments and public institutions could encourage the implementation of the SDGs in the private sector and boost corporate sustainable disclosure initiatives by giving recognition to companies that successfully support the SDGs.

As with all research, our paper has some limitations that suggest future research avenues. A first limitation is related to the methodology since the key words chosen for the article selection exclude studies that target specific SDGs, focusing rather on the whole set of goals. To this effect, studies on the implementation of specific SDGs could be undertaken. Furthermore, only English-language peer-reviewed articles were collected, with a cut-off date of February 2022. Given that the field is evolving rapidly, this paper may not have considered noteworthy articles published after this date and in other languages, so we encourage researchers to continue work in this area, following the guidelines in our Future Agenda section. Last, the paper does not cover the enablers that may exist in the public sector, which is likewise a key actor in the implementation of the goals (Soberón et al. 2020), and the importance of enablers for the policymakers that are challenged to implement the SDGs (Allen et al. 2018). Therefore, the study of enablers for SDG implementation from the point of view of the public sector is advised.

While focusing on the aspects that can enable SDG implementation both in the private and the public sectors is a necessary study topic, it also raises the question of the existing barriers for SDG implementation. We therefore recommend researching the opposite to enablers since understanding the barriers will likely lead to more thorough and accurate information on the topic. Detecting the consequences of SDG implementation for firms, both in the short and the long terms, may also be an interesting line, focusing on the effect on corporate environmental responsiveness (Bhatt & Ghuman 2022) and the positive and negative effects of the introduction of the goals and their subsequent alignment to business practices. Some examples to this effect are employee satisfaction, talent retention, better financial results, more customer engagement and customer relationship management (Müller 2014), and higher reputation, among others. Having identified the limitations, the value of our contribution nonetheless lies in the thoroughness of the analysis performed and the relevance of the studied topic.

All in all, it is clear from the review that integrating the values of Agenda 2030 challenges traditional sustainability models, while aiding businesses in developing sustainability strategies with global aspirations. The SDGs are key in the paradigm of sustainable development, reflecting a balanced approach to economic, social and environmental priorities. For companies in all countries, attaining the SDGs can be a learning mechanism as well as a disruptive transformation, thereby demanding excellence in the process design and management know-how (Yiu & Saner 2017). The key to success in implementing the SDGs (like with any kind of organizational change) is active leadership, above average governance practices and both internal and external communication. Undoubtedly, another key to success is the knowledge of existing enablers for the implementation of the SDGs, which this paper tackles, in line with the recommendations of the professional and academic communities.

The use of the enablers identified in combination with the future agenda proposed, which needs to be sped up, paves the way to promote the SDGs successfully in the private sector. This will be a stepping-stone for the UN’s 2030 Agenda to be accomplished globally in due time. By the end of 2022, the SDG initiative will already be at the half-way point of the 2030 target implementation time frame. Beyond any doubt, time is not in our favour, so immediate action must be taken by all parties.

## Data Availability

Data sharing not applicable to this article as no datasets were generated or analyzed during the current study.

## Appendix 2: Collection of articles included in the review

**Table:**

Cluster	Articles included in the review
Green cluster: Guidelines for sustainable development and achievement of SDGs	(Bhullar 2019; Chan et al. 2020; Dick-Forde et al. 2020; Dobrowolski & Sułkowski 2020; Fleming et al. 2017a, b; Mio et al. 2020; Palmer 2020; Rashed & Shah 2021; Sachs et al. 2019; van Tulder et al. 2021; Yunus et al. 2021)
Blue cluster: International evidence of SDG implementation in different sectors and firm types	(Barkemeyer & Miklian 2019; Buhmann et al., 2019b; Bull & McNeill 2019; Castillo-Villar 2020b; Eweje et al. 2020; Göbel et al. 2017; Grainger-Brown & Malekpour 2019; Gunawan et al. 2020; Gusmão Caiado et al. 2018; Hajer et al. 2015; Imaz & Eizagirre 2020; Jayaprakash & Radhakrishna Pillai 2018; Joseph & Kulkarni, 2020; Maher & Buhmann 2019; Muff et al. 2018; Ordonez-Ponce et al. 2021; Pohlmann et al. 2020; Sun et al. 2020; Wynn & Jones 2019; Zemanová & Druláková, 2020)
Red cluster: Management education for a sustainable development	(Albert & Uhlig 2021; Aleixo et al. 2021; Ali et al. 2018; Alm et al. 2021; Anderson & Ratiu 2019; Beyne 2020; Bremermann et al. 2019; Caldera et al. 2018; Camodeca & Almici 2021; Ferreira Caldana et al. 2022; de Amorim et al. 2020; Fagerlin et al. 2019; Fonseca & Carvalho 2019a, b; Fowler & Biekart 2017; Gil-Doménech et al. 2021; Javeed et al. 2021; Khaled et al. 2021; Khalique et al. 2021; Kolk 2016; Kolk et al. 2017; Leal Filho et al. 2021; Liou & Rao-Nicholson 2021; Liu et al. 2021; Macht et al. 2020; Maslova 2020; Mattera et al. 2021; Montiel et al. 2021; Nishitani et al. 2021; Olfe-Kräutlein 2020; Pizzutilo & Venezia 2021; Rena et al. 2022; Reynolds et al. 2018; Rosati & Faria 2019; Santos et al. 2021; Sierra & Suárez-Collado 2021; Singh & Pathak 2020; Son-Turan 2021; Spallini et al. 2021; Spangenberg 2017; Sree Kumar et al. 2020a, b; Westerman 2021; Westerman et al. 2021; Yamane & Kaneko 2022; Zanellato & Tiron-Tudor 2021a, b)
Yellow cluster: Innovative opportunities to achieve sustainable development in the near future	(Ahmed et al. 2021; Bukalska et al. 2021; Calabrese et al. 2018; Christ & Burritt 2019; Cordova & Celone 2019; D’Amato et al. 2020; Dantas et al. 2021; DeBoer et al. 2020; Govindan et al. 2020; Habib & Chimsom 2019; Morioka et al. 2018; Muntean et al. 2021; Nakidien et al. 2021; Nasution et al. 2020; Nayal et al. 2021; Saner et al. 2020; T. Santos et al. 2021; Taminiau et al. 2017; Walker et al. 2021; Yiu & Saner 2017)
